# Elevated leptin levels in healthy climacteric women from Northeastern Brazil: an effect of age or adiposity?

**DOI:** 10.20945/2359-3997000000249

**Published:** 2020-06-05

**Authors:** Ana Cyntia B. N. Maniçoba, Clariano P. Oliveira, Johnny. R. Nascimento, Flávia R. F. Nascimento, Haissa O. Brito, Rui Miguel Gil da Costa, Maria do Carmo L. Barbosa, Manuel dos Santos Faria, Maria do Desterro S. B. Nascimento, Luciane M. O. Brito

**Affiliations:** 1 Programa de Pós-Graduação em Saúde do Adulto Universidade Federal do Maranhão São Luís MA Brasil Programa de Pós-Graduação em Saúde do Adulto, Universidade Federal do Maranhão (UFMA), São Luís, MA, Brasil; 2 Faculdade de Medicina Universidade Federal do Maranhão São Luís MA Brasil Faculdade de Medicina, Universidade Federal do Maranhão (UFMA), São Luís, MA, Brasil; 3 Departamento de Ginecologia Hospital Universitário Universidade Federal do Maranhão São Luís MA Brasil Departamento de Ginecologia, Hospital Universitário, Universidade Federal do Maranhão (UFMA), São Luís, MA, Brasil; 4 Programa de Pós-Graduação em Ciências da Saúde Universidade Federal do Maranhão São Luís MA Brasil Programa de Pós-Graduação em Ciências da Saúde, Universidade Federal do Maranhão (UFMA), São Luís, MA, Brasil; 5 Departamento de Endocrinologia Hospital Universitário Universidade Federal do Maranhão São Luís MA Brasil Departamento de Endocrinologia, Hospital Universitário, Universidade Federal do Maranhão (UFMA), São Luís, MA, Brasil

**Keywords:** Metabolic syndrome, menopause, leptin, climacterium, inflammation

## Abstract

**Objective:**

Climacterium is associated with elevated leptin levels and increased risk of cardiovascular disorders. Conflicting data diverge on whether high leptin levels in climacterium reflect increasing adipose mass or, at least partially, age-related hormonal changes. This study addresses this issue in women from a Brazilian state with a low human development index.

**Subjects and methods:**

A case-control study was conducted, enrolling 136 women from the state of Maranhão, 52 (38.2%) climacteric and 84 (61.8%) non-climacteric. Biometric, biochemical, hormonal and immunological parameters were analyzed.

**Results:**

Climacteric women showed a moderately increased waist/hip ratio (0.894 versus 0.834, p < 0.05), sustained body mass index (27.46 versus 28.68, p > 0.05) increased leptin levels (9.59 versus 7.13, p < 0.05) and no evidence of metabolic syndrome. No other parameters were altered. The climacteric cohort didn’t show significant body fat gains but displayed a typical age-related redistribution of adipose tissue. Even so, leptin levels were significantly elevated compared with non-climacteric women.

**Conclusions:**

Altogether, these data support the hypothesis that leptin is elevated, at least partially, as a function of age and climacterium and is not necessarily correlated with metabolic dysfunction and systemic inflammation. Further studies are needed to evaluate the impact of higher leptin levels on postmenopausal women. Arch Endocrinol Metab. 2020;64(3):276-81

## INTRODUCTION

During climacterium there is a redistribution of body fat with a tendency for adipose tissue to accumulate in the abdominal region, as previously reviewed ( 1 ). This redistribution is associated with profound changes in energy metabolism and may also include a significant total weight gain and obesity ( 2 ). Obesity is associated with a chronic state of low-grade inflammation presenting increased levels of inflammatory markers, such as C-reactive protein (CRP) and pro-inflammatory cytokines ( 3 ). All these changes increase the risk of some types of cancer, like breast cancer, which are promoted by obesity and chronic inflammation ( 4 , 5 ). The lack of ovarian hormones also increases the risk of developing cardiovascular disorders, and some hormonal replacement therapies show encouraging results in preventing coronary arterial disease ( 6 ). In fact, the risk of developing metabolic syndrome and diabetes in considerably increased by the climacterium ( 7 ).

White adipose tissue consists of preadipocytes and adipocytes, macrophages, endothelial cells, fibroblasts and leukocytes, and secretes a variety of regulatory molecules, known as adipokines, which are involved in regulating body functions like the metabolism of glucose and fatty acids, energy expenditure, inflammatory responses, immunity, cardiovascular function and reproduction ( 2 ). Leptin is a well-known adipokine involved in energy metabolism and reproductive function, and plasma leptin levels vary directly according to body mass index (BMI) and body fat percentage ( 8 ). Subcutaneous adipose tissue secretes a greater amount of leptin than visceral adipose tissue ( 9 , 10 ). Leptin acts over hypothalamic neurons to reduce appetite and the development of obesity in aging climacteric women has been proposed to consist on a state of leptin resistance ( 11 ). Hypothalamic leptin receptors co-localize with estrogen receptor alpha (ERα), suggesting that the lack of estrogenic stimulation in climacteric women may contribute to leptin resistance ( 12 ). Furthermore, supplementation with exogenous estrogen in ovariectomized female rats restored leptin sensitivity and normalizes the distribution of adipose tissue ( 13 ). Although high leptin levels are associated with increased BMI and other metabolic risk factors, data from climacteric women receiving hormonal replacement therapy do not show a consistent improvement on circulating leptin levels or its activity ( 5 ). However, data from climacteric women receiving hormonal replacement therapy do not show a consistent improvement on circulating leptin levels or its activity ( 14 , 15 ). In fact, previous reports suggest that leptin levels in women are more related to adiposity than to age and age-related hormonal changes ( 16 ).

The present study compared leptin serum levels, endocrine and immunologic profiles and biometric markers of metabolic risk between climacteric and pre-climacteric women.

## SUBJECTS AND METHODS

### Patients

All procedures were in accordance with the ethical standards of the National Health Council resolution number 466/12 and the 1964 Helsinki declaration and its later amendments. This research project was approved by the Research Ethics Committee of the Federal University of Maranhão with approval number 698.706, in accordance with the National Health Council resolution number 466/12. All participating women were informed about the research and signed an informed consent form.

Climacteric (case) and non-climacteric (control) women visiting the Climaterium Reference Service of the Federal University of Maranhão University Hospital (HUUFMA) were included. Inclusion criteria for the case group included women with 35-65 years and clinically characterized as being in the climacteric period due to the occurrence of menopause or presence of symptoms of hypoestrogenism ( *e.g.,* menstrual changes, hot flashes, emotional lability) for at least 12 months. The control group was composed of age-matched non-climacteric women. For both groups, exclusion criteria were: patients undergoing hormonal replacement therapy and patients undergoing cancer treatment. In total, 136 women were included in the study, 52 (38.2%) in the climacteric group and 84 (61.8%) in the non-climacteric group.

### Sociodemographic variables

All patients completed a questionnaire concerning their age, profession, marital status and educational level.

### Biometric measurements

The women were weighed using an electronic scale (BL2096PP, Toledo) with a weighing capacity of up to 200 kg and a precision of 100 g. For weighing, women were guided according to Jelliffe’s criteria. The height (cm) was determined using a vertical anthropometer, which was coupled to the electronic scale ( 17 ). The Body Mass Index (BMI) was calculated by dividing weight by height^2^(kg/m^2^). The waist circumference (WC) was measured at the smallest circumference between the last rib and the iliac crest using an inelastic tape measure without exerting compression on the tissues. The waist/hip ratio (WHR) was obtained by dividing the value of the waist circumference by the value of the hip circumference (both in cm). All cut-off points were those recommended by the WHO ( 18 ).

### Blood biomarkers

All participants were invited to collect blood samples. All women were instructed to fast for 12 hours, including not drinking alcohol for 72 hours, as well as not performing intense physical activities 24 hours before the test. The evaluation of the lipid profile comprised HDL, LDL, VLDL, triglycerides and total cholesterol. The hormonal profile included follicle-stimulating hormone (FSH), luteinizing hormone (LH), estradiol and progesterone. For pre-menopausal women, samples were collected on the third day of the cycle.

The concentrations of leptin, IL-2, IL-4, IL-6, IL-10, TNF, IFN-γ and IL-17a were determined by an enzyme-linked immunosorbent assay (ELISA) technique, using R&D Systems Quantikine ELISA Kits (R&D Systems Minneapolis, MN) according to manufacturer’s specifications. All dosages were determined in duplicate. The results were presented in pg/mL.

### Statistical analysis

A computer database was created using SPSS, version 22.0. Chi-square, Fisher’s exact test and OR (95% CI) were used for analyzing qualitative variables between the groups. For multivariate analysis, the Logistic Regression model was used. For the quantitative variables with a normal distribution, the mean, standard deviation, minimum and maximum values, or the median, were calculated. For variables with an asymmetric distribution, the percentiles 25 and 75 were calculated. The statistical tests used were Student’s *t* test for independent samples for quantitative variables with a normal distribution, or the Mann-Whitney test for variables with an asymmetric distribution. A significance level of *p* < 0.05 was adopted.

## RESULTS

We evaluated 136 women, 52 (38.2%) of which were climacteric patients and 84 (61.8%) were non-climacteric patients. We divided the patients into two age groups: 35-49 years-old and 50-65 years-old, and confirmed that climacteric women predominantly belonged in the older group (54 ± 7.08 years-old), while non-climacteric women predominantly belonged to the younger (43 ± 5.65 years-old) group ( Table 1 , *p* < 0.001). We found no statistically significant difference when women were grouped according to their marital status. However, we observed marginally significant differences when women were grouped according to their educational level ( *p* < 0.048) and their profession ( *p* < 0.045). Non-climacteric women tended to have a higher educational level then climacteric women and to be employed, while climacteric women more frequently stayed at home as housewives.

**Table 1:** Socio-demographic characteristics (mean, standard deviation, maximum and minimum values) in climacteric women, São Luís, Maranhão, Brazil

	Climacterium				

	Yes (52)		No (84)		p

	n	%	n	%	
Age group					0.001
35-49 years	10	19.2	66	78.6	
50-65 years	42	80.8	18	21.4	
Marital status					0.449
Single	25	49.0	32	38.1	
Married/Cohabitation	18	35.3	40	47.6	
Separated/Divorced	5	9.8	9	10.7	
Widow	3	5.9	3	3.6	
Education level					0.048
Illiterate	2	3.9	0	0.00	
Complete Elementary School	9	17.6	9	10.7	
Incomplete Elementary School	16	31.4	21	25.0	
Complete High School	17	33.3	39	46.4	
Incomplete High School	4	7.8	7	8.3	
Incomplete Higher Education	3	5.9	7	8.3	
Complete Higher Education	0	0	1	1.2	
Profession					0.045
Housemaid	7	13.5	4	4.8	
Housewife	16	30.8	15	17.9	
Employee	17	32.7	36	42.9	
Others	12	23.1	29	34.5	

Concerning morphometric variables, the climacteric group showed increased WHR ( *p* = 0.031) compared with the non-climacteric group, although there were no changes concerning the BMI ( Table 2 ). As expected, the climacteric group showed much lower estradiol and progesterone levels compared with the non-climacteric group, while FSH and LH values were higher ( Table 3 , *p* < 0.001). Additionally, climacteric women showed higher blood levels of triglycerides, LDL, VLDL and total cholesterol, but these changes did not reach statistical significance.

**Table 2:** Biometric variables (mean, standard deviation, maximum and minimum values) in patients from São Luís, Maranhão, Brazil

	Climacterium								

	Yes				No				p
	
	Mean	SD	Max	Min	Mean	SD	Max	Min	
BMI	27.46	6.52	37.16	2.25	28.68	4.58	38.24	20.7	0.507
Height	1.52	0.04	1.62	1.44	1.55	0.06	1.66	1.46	0.055
Weight	65.7	12.1	90.7	42.6	68.8	11	90.7	49.1	0.383
WHR	0.894	0.09	1.06	0.75	0.834	0.083	1.03	0.73	0.031

SD: standard deviation; Max: maximum; Min: minimum; H/W: hip/waist ratio.

**Table 3:** Biochemical and immunological markers (mean, standard deviation, maximum and minimum values) in climacteric women, São Luís, Maranhão, Brazil

	Climacterium								

	Yes				No				p
	
	Mean	SD	Max	Min	Mean	SD	Max	Min	
Estradiol (pg/ml)	23	45.1	284.9	2.5	156	154.9	545.7	2.5	<0.001
FSH mIU/mL	64.4	32.9	134.6	1.1	19.6	25.1	83,9	1.8	<0.001
LH (mIU/mL)	28.26	12.64	56.8	0.45	16.01	15.45	50.77	1.13	<0.001
Progesterone (ng/mL)	0.375	0.358	2.49	0.103	4.696	6.063	19.16	0.026	<0.001
HDL (mg/dL)	49.1	12.1	80	28	50.5	9	66	37	0.523
LDL (mg/dL)	134.7	34.9	218	51	118.8	39.1	202	56	0.093
Fasting glucose (mg/dL)	108.3	53	346	77	114	55.9	281	80	0.379
Triglycerides (mg/dL)	141.2	71.9	413	48	113.8	59.5	262	43	0.087
VLDL (mg/dL)	28.3	14.4	83	10	22.8	11.8	52	9	0.094
CRP (mg/dL)	0.287	0.359	1.24	0.01	0.246	0.419	1.23	0.01	0.251
Total cholesterol (mg/dL)	212.1	36.6	291	137	192	44.9	297	125	0.046
Leptin	9.59	6.3	17.84	0.25	7.13	5.36	17.85	0.33	0.03
IL 2	7.9	40.6	270	0.21	22.5	148.2	1,146	1.19	0.329
IL 4	0.2	1.1	8	0.03	17.5	122.3	1,067	0.47	0.387
IL 6	91.9	138.5	585	0.17	73.8	138.1	771	0.22	0.174
IL 10	251.1	1,684.5	12,161	0.07	38.9	187.1	1,429	0.12	0.216
TNF	13.2	66.8	450	0.64	28.2	189.3	1,532	1.73	0.329
IFNy	3.3	16.1	83	0.04	8.7	61.6	530	0.83	0.329
IL 17a	185.4	554.4	3,218	3.42	143.9	602.3	5,121	4.26	0.693

SD: standard deviation; max: Maximum; min: Minimum.

Importantly, the climacteric group showed significantly higher leptin levels compared with the non-climacteric group ( Table 3 , *p* = 0.030). There were no significant differences concerning the inflammatory marker CRP or any of the pro-inflammatory and anti-inflammatory cytokines tested.

## DISCUSSION

In women, the onset of climacterium is associated with a number of changes with adverse health impacts, ranging from diabetes and metabolic syndrome to cardiovascular disorders and some types of cancer. The present show that climacteric women with a moderate increase in their waist to hip ratio (WHR) but no increased BMI or biochemical markers of metabolic dysfunction already show increased leptin levels, compared with pre-climacteric women. The accumulation of adipose tissue and its redistribution from subcutanous sites into visceral abdominal fat is associated with higher circulating levels of cholesterol and triglycerides. These alterations greatly increase the risk of atheromatous changes in the arterial wall and leading, e.g. to coronary artery disease ( 19 ). Climacterium is also associated with a state of chronic systemic inflammation. Inflammatory mediators released by accumulated visceral fat tissue contribute for enhancing vascular damage. Accumulating evidence also shows that chronic systemic inflammation promotes several types of cancer, like breast and colon cancer ( 4 - 5 , 20 ). The circulating levels of adipokines like leptin and adiponectin have been suggested as potential markers of increased risk for developing cardiovascular disorders ( 21 ) and breast cancer ( 22 ) but their potential role in the pathogenesis of these disorders remains obscure. The most relevant factor responsible for up-regulating leptin following climacterium is also a matter of debate, with some studies implicating the lack of ovarian hormones ( 13 ), while others suggest that higher leptin levels simply reflect increased adipose mass ( 15 , 16 ). Schautz and cols. ( 16 ) suggest that advancing age has a lesser but independent effect on leptin levels, compared with adiposity.

The present data indicate that circulating leptin levels are significantly increased in climacteric women, even in the absence of a significantly increased BMI or other indicators of obesity or metabolic dysfunction. In this study, climacteric women were, as expected, older than their non-climacteric controls. Interestingly, climacteric women had less advanced education levels and were more likely to work as housewives, while non-climacteric women showed better educational levels and were more likely to have jobs outside home. This probably reflects recent generational changes in Brazilian society, that saw young women gain access to schools and the labor market.

The biometric profile of climacteric and non-climacteric women had some important similarities and differences. Climacteric women and non-climacteric controls showed similar body weight and BMI, ruling out climacterium-associated obesity. Climacteric women did show a significantly increased HWR, reflecting a typical redistribution of body fat into the abdominal area. This is in contrast with data obtained from biochemical blood analyses, showing that cholesterol, glucose and triglycerides levels weren’t significantly higher in climacteric women compared with non-climacteric controls. These data suggest our study population predominantly included healthy climacteric women, who may or may not evolve to develop metabolic disorders in the future. Importantly, leptin levels were significantly elevated in climacteric versus non-climacteric women, even in the absence of significant biometric or biochemical changes. This lends support to the hypothesis that increased leptin levels in climacteric women are not solely driven by the accumulation of adipose tissue, but are also influenced by other factors like the loss of ovarian hormones. It is tempting to speculate that, during climacterium, the loss of ovarian hormones may induce an early, moderate but significant increase in leptin levels, while obesity further increases leptin levels chronically over time.

Climacterium is also associated with a status of chronic inflammation and pro-inflammatory cytokines, associated with an increased risk of cardiovascular disease, are often up-regulated in climacteric women ( 2 , 23 ). Leptin is associated with this pro-inflammatory status and has been proposed to directly contribute for leukocytic differentiation and cytokine production ( 24 ). In the present study, there were no significant differences concerning C reactive protein nor any of the cytokines tested. This may reflect the fact that our cohort of climacteric women didn’t show obesity, since the adipose tissue is known to produce many of these inflammatory mediators ( 2 ).

In this study, there was no opportunity to determine whether the increased leptin levels observed in climacteric women may be predictive of certain outcomes (i.e., do climacteric women with higher leptin levels tend to develop obesity, or metabolic syndrome, or cardiovascular diseases or certain types of cancer). This important issue may be addressed by additional prospective studies.

Overall, the present results show that high leptin levels may be found in climacteric women in the absence of an elevated BMI or other signs of obesity. This urges caution in the interpretation of leptin levels in this population. Additionally, these findings suggest that leptin up-regulation may be driven, at least in part, by the loss of ovarian hormones.
